# Patients’ and Providers’ Needs and Preferences When Considering Fertility Preservation Before Cancer Treatment: Decision-Making Needs Assessment

**DOI:** 10.2196/25083

**Published:** 2021-06-07

**Authors:** Aubri Hoffman, Laura Crocker, Aakrati Mathur, Deborah Holman, June Weston, Sukhkamal Campbell, Ashley Housten, Andrea Bradford, Shilpi Agrawala, Terri L Woodard

**Affiliations:** 1 Department of Gynecological Oncology and Reproductive Medicine The University of Texas MD Anderson Cancer Center Houston, TX United States; 2 Department of Health Services Research The University of Texas MD Anderson Cancer Center Houston, TX United States; 3 Department of Biostatistics The University of Texas at Arlington Arlington, TX United States; 4 Department of Obstetrics and Gynecology Baylor College of Medicine Houston, TX United States; 5 Department of Medicine Baylor College of Medicine Houston, TX United States

**Keywords:** cancer, decision support techniques, fertility preservation, oncofertility, oncology, needs assessment, patient decision aids, patient needs, shared decision making

## Abstract

**Background:**

As cancer treatments continue to improve, it is increasingly important that women of reproductive age have an opportunity to decide whether they want to undergo fertility preservation treatments to try to protect their ability to have a child after cancer. Clinical practice guidelines recommend that providers offer fertility counseling to all young women with cancer; however, as few as 12% of women recall discussing fertility preservation. The long-term goal of this program is to develop an interactive web-based patient decision aid to improve awareness, access, knowledge, and decision making for all young women with cancer. The International Patient Decision Aid Standards collaboration recommends a formal decision-making needs assessment to inform and guide the design of understandable, meaningful, and usable patient decision aid interventions.

**Objective:**

This study aims to assess providers’ and survivors’ fertility preservation decision-making experiences, unmet needs, and initial design preferences to inform the development of a web-based patient decision aid.

**Methods:**

Semistructured interviews and an ad hoc focus group assessed current decision-making experiences, unmet needs, and recommendations for a patient decision aid. Two researchers coded and analyzed the transcripts using NVivo (QSR International). A stakeholder advisory panel guided the study and interpretation of results.

**Results:**

A total of 51 participants participated in 46 interviews (18 providers and 28 survivors) and 1 ad hoc focus group (7 survivors). The primary themes included the importance of fertility decisions for survivorship, the existence of significant but potentially modifiable barriers to optimal decision making, and a strong support for developing a carefully designed patient decision aid website. Providers reported needing an intervention that could quickly raise awareness and facilitate timely referrals. Survivors reported needing understandable information and help with managing uncertainty, costs, and pressures. Design recommendations included providing tailored information (eg, by age and cancer type), optional interactive features, and multimedia delivery at multiple time points, preferably outside the consultation.

**Conclusions:**

Decision making about fertility preservation is an important step in providing high-quality comprehensive cancer care and a priority for many survivors’ optimal quality of life. Decision support interventions are needed to address gaps in care and help women quickly navigate toward an informed, values-congruent decision. Survivors and providers support developing a patient decision aid website to make information directly available to women outside of the consultation and to provide self-tailored content according to women’s clinical characteristics and their information-seeking and deliberative styles.

## Introduction

With cancer survival rates exceeding 75% for young adults, it is increasingly important to minimize the negative effects of treatment, such as infertility, to protect survivors’ long-term quality of life. Infertility ranks fifth on the World Health Organization’s list of serious disabilities, and multiple studies show that cancer-related infertility causes distress and regret that persist long term, even in survivors with otherwise positive mental health status [1-4]. Since 2006, the American Society of Clinical Oncology (ASCO) guidelines have recommended that oncology providers address the risk of treatment-related infertility as early as possible and offer all women a referral for fertility counseling before initiating cancer treatment [5-7]. The decision-making process consists of 2 steps: patients must decide whether to accept or seek a referral to a fertility specialist and then decide whether to undergo fertility preservation and of which type. Referral to a fertility specialist decreases survivors’ long-term regret and distress, whether or not women undergo fertility preservation therapies [2,3]. However, a recent review reported that 25% of physicians, 36% of advanced practice nurses, and 65% of nurses are not aware of the guidelines [8,9]. Only 53% of women of reproductive age recalled having a discussion about the effect of cancer treatments on fertility, and as few as 12.2% women recalled having fertility preservation counseling [2,10].

Several previous studies have explored barriers to fertility preservation discussions, referrals, and treatment [11-15]. Oncology providers report a lack of time for counseling within the consultation and resources to offer patients, particularly those who do not have insurance coverage. Provider education interventions and financial resource programs have been developed to address these barriers. However, gaps remain in helping providers also identify appropriate patients for referral, which necessitates shared decision making to assess patients’ informed personal preferences [8]. For patients, educational booklets have improved awareness and information comprehension; however, gaps remain in providing evidence-based decision support during the brief time (sometimes as little as 24 hours) between diagnosis and cancer treatment initiation [8,16-18]. Interventions are needed that can provide timely information and decision support to women when they need it and can process it, which is often in between clinical consultations.

Patient decision aids are tools that complement clinical consultations by providing up-to-date, plain language descriptions of the options as well as theory- and evidence-based approaches to help patients engage in decision making [19-21]. They are designed for preference-sensitive decisions in which there are 2 or more medically appropriate options, high uncertainty, and trade-offs in risks and benefits [22]. Decision aids may be provided in a variety of formats (eg, booklets, videos, and websites) before, during, or after a clinical consultation. The most recent Cochrane Collaboration review of 89 randomized trials reported that patient decision aids improve patients’ knowledge, realistic expectations of risks, and congruency of treatment choices with their informed *decision-making values* (ie, the factors in the decision that matter most to the patient) [19]. Studies show that patient decision aids improve decision quality (ie, the degree to which decisions are well informed, congruent with what patients value most, and actionable) and reduce decisional conflict (a state of anxiety that blocks action) [19,23,24]. Decisional conflict has been shown to correlate with long-term decisional regret [24]. For every unit increase in decisional conflict, patients are 59 times more likely to change their mind, 23 times more likely to delay, 3 times more likely to fail knowledge tests, and 19% more likely to blame the doctor for bad outcomes [24-26].

In Australia [27], the Netherlands [28], Germany [29], and the United Kingdom [30], fertility preservation patient decision aids have been developed, with positive results on knowledge, decisional conflict, and decisional regret. However, at the time of this study, these decision aids were only available for women with breast cancer. Furthermore, many women in the United States face an additional financial barrier—as of August 2020, only 19 states mandate insurance coverage for fertility counseling or care (with only 10 states covering iatrogenic fertility loss), and few women are aware of available financial resources such Walgreens Heartbeat and LIVESTRONG Fertility [10,16,31]. The long-term goal of this program of research studies is to develop a patient decision aid [32] that provides information tailored for a variety of cancer types and time points and that specifically addresses women’s decision-making, financial, and referral and navigation needs.

As a key step in the systematic development of a patient decision aid, the International Patient Decision Aid Standards (IPDAS) Collaboration recommends a formal assessment of all stakeholders’ decision-making needs and perspectives to ensure that the decision aid will be effective, meaningful, usable, and sustainable [33-36]. 

Following the Decisional Needs Assessment approach [37], the specific aims of this study are to assess the decision-making needs of oncology providers and female cancer survivors in terms of their (1) current experiences and key decision-making factors, (2) unmet decision-making needs, and (3) recommendations for the content and design of a fertility preservation patient decision aid website.

## Methods

### Conceptual Framework

**Figure 1:**
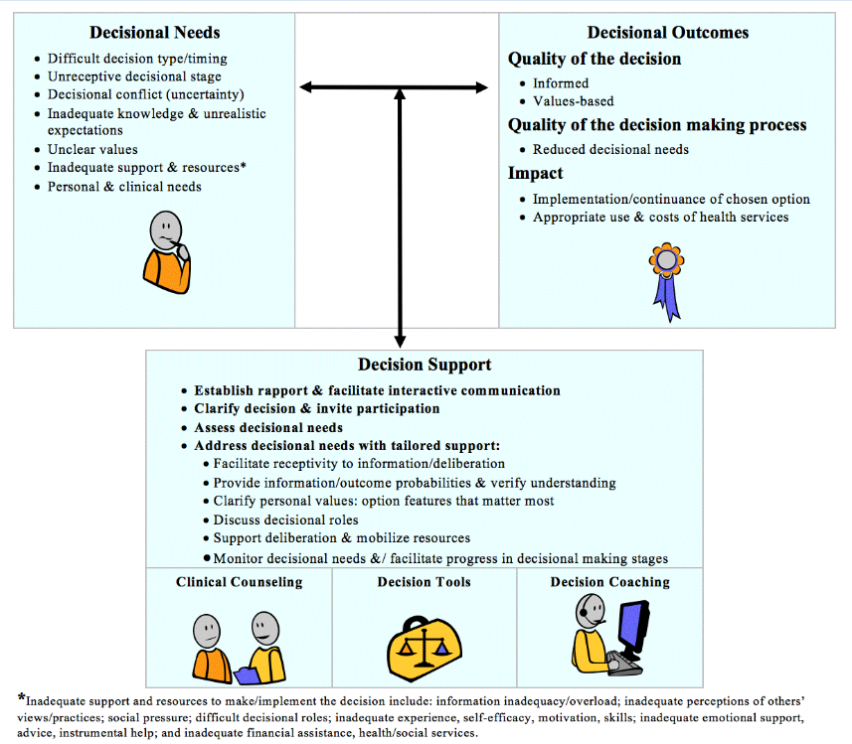
The Ottawa Decision Support Framework.

The Ottawa Decision Support Framework (Figure 1) [38] has been used to develop decision support interventions in more than 100 studies across 18 countries. This framework applies behavioral economic [39,40] and cognitive [39,41-43] theories to preference-sensitive health care decisions. For example, it postulates several modifiable decision support needs, such as lack of awareness, knowledge, clarity, or support, which may be addressed to ensure a high-quality decision-making process. The framework was operationalized in 1999 in the Decisional Needs Assessment Workbook (updated in 2013 [37]), which details a series of 10 steps to define and select the appropriate objective(s), participants (key informants), rationale, methods, data collection tools, sample size, sampling procedure, analytic methods, and presentation of findings. The research team completed these steps, resulting in the approach described here in the *Methods* section.

### Study Design

**Figure 2:**
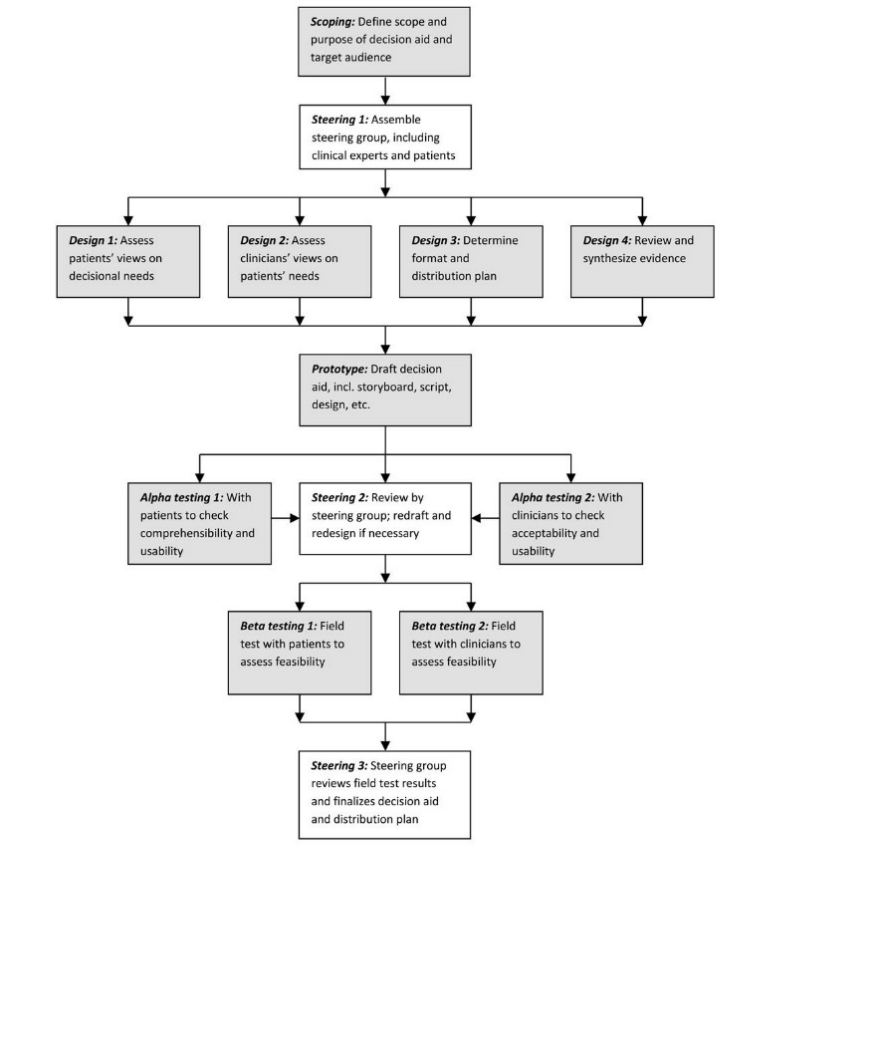
The International Patient Decision Aid Standards Collaboration model for systematic development of a patient decision aid.

To address the specific aims, this study used semistructured cognitive interviews and an ad hoc semistructured focus group. Figure 2 presents the IPDAS model [33] for rigorous and systematic patient decision aid development of patient decision aids. This study specifically addressed design steps 1 and 2. Previously, we engaged a stakeholder advisory panel of female cancer survivors, patient advocates, and oncology providers who guide the larger program of research. The advisory panel actively partners in each study, including contributing to the study design, protocol, materials, instruments, interpretation of results, presentations, and manuscripts. For this study, the advisory panel and research team defined the scope of this needs assessment to focus on assessing women with cancer’s needs for information and decision support regarding whether to seek or accept a referral for fertility counseling as well as exploring whether an interactive website would be feasible. These decisions were based on 3 key factors: (1) previous studies had assessed decision making about choosing among preservation options but not the initial choice to seek or accept the referral to see a fertility specialist; (2) given the short time frame, survivors report needing access to information between consultations at all hours; and (3) a website could provide interactive features to allow self-tailoring by cancer type (eg, breast and ovarian), age, information level, deliberative style, or other factors. Subsequent planned studies will assess technological aspects, such as accessibility, usability, design, and acceptability, to create an optimized prototype for pilot testing. The MD Anderson Cancer Center Institutional Review Board provided ethical review and approval for this study (#2014-0130).

### Participants: Oncology Providers and Female Cancer Survivors

The principal investigator (TLW) purposefully invited oncology providers at the institution representing a variety of professional roles (surgeons, advance practice providers, etc), clinical expertise, experience, and sociodemographic characteristics. To recruit survivors, the research team distributed flyers in the waiting areas and lobbies throughout the MD Anderson Cancer Center and community clinics and invited survivors in person after their appointments at the Oncofertility Clinic. Over the past 10 years, the racial and ethnic distribution of survivors seen at the Oncofertility Clinic included 65% White, 11.6% Black, 10.5% Asian, 5.7% other, and 22.5% Hispanic individuals (7.2% declined to respond). Eligible survivors included English-speaking 18- to 45-year-old females diagnosed with cancer within the previous 5 years who received a potentially fertility-damaging treatment (eg, chemotherapy, radiation, or surgery on a reproductive organ). In the context of this sensitive survivorship topic, it is important to note that *women* is the inclusive term preferred by our survivors, as fertility counseling includes offering fertility preservation treatments and/or alternative family-building options to patients and survivors who may or may not currently have a uterus or ovaries. All participants provided informed consent.

### Interviews

Following the Decisional Needs Assessment Workbook [37], the interview guide (Multimedia Appendix 1) asked providers and survivors to reflect on (1) their current experiences, (2) unmet needs, and (3) recommendations for the content and design of a patient decision aid. The Decisional Needs Assessment in Populations Workbook [37] provides templates of questions that may be readily adapted as appropriate for the clinical context and user(s). The advisory panel also edited questions for patient-centered language. For example, we tailored the questions to focus on referrals for fertility counseling and for survivors or providers (eg, What factors do you consider and/or discuss with patients to decide whether to refer for fertility? and What types of things were you thinking about when deciding whether to go see the fertility specialist?). The principal investigator and a trained research coordinator conducted the cognitive interviews, which were audio recorded using integrated overhead microphones and transcribed verbatim. Women also completed a questionnaire assessing their characteristics and ratings of the helpfulness of potential medium and delivery formats (to confirm, or add to, plans for web-based delivery). At the end of the interview, women received a US $50 gift card and paid parking. Both interviewers took field notes, which were compared and discussed to inform the interpretation of results. Recruitment continued until data saturation was reached, in terms of no new codes emerging in 2 cycles of transcript coding (ie, 2 batches of 2 to 3 transcripts).

### Focus Group

While analyzing the cognitive interview transcripts, the research team observed that participants had introduced ideas that merited further exploration, including a variety of factors that drove their decisions (ie, their *decision-making values* [44]), and a variety of resources that they used during their decisions. Decision-making values are a key component of decision support, and many patient decision aids present a set of top-ranked values to engage patients in considering which factors are most important to them in this decision (ie, decision-making values clarification). After discussion with the advisory panel, the principal investigator and research assistant conducted an ad hoc survivor focus group to confirm coding and interpretation (including which decision-making factors were top priority) and to assess responses to relevant resources. The research assistant recontacted 2 individuals from the interviews who had made statements that merited additional discussion and recruited 5 additional survivors. Following the focus group discussion guide (Multimedia Appendix 2), the principal investigator invited women to complete an initial questionnaire that presented women with a set of selected values statement from the interview transcripts. Women ranked the importance of each statement individually, discussed their ranking as a group, and added additional ideas they felt were not yet well addressed. Next, the research assistant presented the group with the cancer center’s patient information sheets, the existing patient decision aids [27-29], and examples of the web-based resources that interview participants had recommended. The research assistant used open-ended discussion prompts to assess women’s perspectives on the wording, graphic design, utility, acceptability, and usefulness. Participants were provided with US $50 gift cards and paid parking.

### Data Analysis

Interviews were coded and analyzed using the coding process by Strauss and Corbin [45] and NVivo 10 (2016 QSR International). Two research assistants coded the first 5 transcripts to create the codebook and then iteratively until saturation was reached (ie, no additional codes were identified in the next 2 transcripts). Each coder separately coded the remaining interviews in batches of 2 to 3. Additional codes were added, and the earlier interviews were reanalyzed and updated. A third coder rereviewed all transcripts to ensure fidelity to the codebook and to ensure that interrater reliability remained above 90%. The principal investigator resolved minor coding discrepancies through discussion and independent review of the transcripts.

Interview findings were summarized using an initial word frequency analysis, followed by keywords in context analysis to identify primary themes and subthemes. There were not sufficient distributions for subgroup analyses; however, exemplary quotes were purposively selected across age and parity subgroups. The research team also compared and contrasted interview transcripts to identify potential missing items or misconceptions that could be addressed in a patient decision aid. As the focus group was conducted to clarify specific points and obtain initial reactions to existing materials, the transcript was analyzed descriptively and the results were summarized in terms of notable points and recommendations that could inform the future design and user testing studies. All findings and interpretations were shared with the stakeholder advisory panel to confirm meaningful interpretations.

## Results

### Participant Characteristics

All 51 individuals who were recruited in person agreed to participate, including 18 oncology providers and 33 female cancer survivors. None of the women responded solely to public flyers. Interview recruitment continued until data saturation (n=46, including 18 providers and 28 survivors). The follow-up focus group reengaged 2 survivors from the interviews and 5 additional survivors (n=7).

Tables 1 and 2 present participants’ characteristics. The average age of survivors was 32 years, and the majority were White, 2 years postdiagnosis, had a college degree, and were in heterosexual relationships without children. The majority of providers were White, female attending physicians with an average of 9 years of clinical experience. Survivors included women seen by participating providers and women seen by other providers.

**Table 1:** Providers’ characteristics (n=18).

Characteristic			Value
**Specialties, n (%)**			
	Medical oncology	1 (6)	
	Surgical oncology	1 (6)	
	Radiation oncology	4 (22)	
	Pediatric oncology	2 (11)	
	Gynecological oncology	3 (17)	
	Lymphoma or myeloma	3 (17)	
	Leukemia	2 (11)	
	Stem cell transplant	2 (11)	
Years in practice, median (range)			9 (3-18)
Female, n (%)			13 (72)
**Race, n (%)**			
	White	8 (45)	
	Black	2 (11)	
	Asian	6 (33)	
	Other	2 (11)	
Ethnicity (Hispanic), n (%)			2 (11)
**Provider type, n (%)**			
	Attending physician	13 (72)	
	Advanced practice provider	5 (28)	

**Table 2:** Survivors’ characteristics (n=33).

Characteristic			Value
**Cancer site, n (%)**			
	Breast	11 (33)	
	Ovarian, cervical, or vaginal	5 (15)	
	Lymphoma or leukemia	14 (42)	
	Other	3 (9)	
Age (years), median (range)			32 (20-45)
Years since diagnosis, mean (range)			2.1 (0-5)
Female, n (%)			33 (100)
**Race, n (%)**			
	White	25 (76)	
	Black	4 (12)	
	Asian	3 (9)	
	Other	1 (3)	
Ethnicity (Hispanic), n (%)			5 (15)
**Highest education, n (%)**			
	Some college	7 (21)	
	Associate degree	4 (12)	
	Bachelor’s degree	15 (46)	
	Master’s degree	6 (18)	
	Professional degree	1 (3)	
**Relationship status at diagnosis, n (%)**			
	Single	10 (30)	
	In a relationship	14 (42)	
	Married	7 (21)	
	Divorced	1 (3)	
	Other	1 (3)	
Nulliparous at diagnosis, n (%)			30 (90)
Completed fertility preservation therapy, n (%)			18 (55)

### Interviews

Interviews and the focus group lasted 1 hour each and were conducted in private rooms at the cancer center. Across all participants, 3 primary themes emerged: (1) fertility preservation decisions are important for survivorship, (2) significant but potentially modifiable barriers to decision making exist, and (3) support exists for a carefully designed fertility preservation patient decision aid. Within the primary themes, 10 subthemes emerged from the 74 unique codes. The following sections describe providers’ and survivors’ comments by subtheme and provide exemplary quotes.

#### Primary Theme 1: Fertility Preservation Decisions Are Important for Survivorship

All participants emphasized the importance of fertility decisions, both for cancer treatment and for long-term survivorship. A total of 3 subthemes were identified (Textbox 1): fertility is important for quality of life during survivorship, knowing the options matters, and fertility desires change over time. The majority of survivors emphasized the importance of knowing that there were options and the benefit of both the discussion process and fertility preservation, if desired, for their overall treatment and recovery. Women who made fertility preservation decisions specifically commented about feeling empowered, hopeful, and/or able to look forward to the future while enduring their cancer treatments. In addition, several survivors described the value of explicitly acknowledging that family-building desires may change over time. Providers also reported the importance of fertility for survivorship. They added concerns about the number of women waiting until after cancer treatment to consider fertility. Several providers discussed the potential of shared decision making to minimize future regret.

**Subthemes and exemplary quotes for primary theme 1 (fertility preservation decisions are important for survivorship).**

**Subtheme 1: Addressing Fertility Is Important**

- “It is really about [having] that conversation. I’ve learned from survivors, that they are: ‘I didn’t know, nobody told me. I have this question mark. I feel like this is cancer all over again.’ It is incertitude that is really...the most burdensome.” (Provider 17, leukemia nurse practitioner)
- “...if this is going to be a long-term cure, we need to look at the whole picture and stop just treating the one aspect of their life. Empowering patients themselves to ask about their risk of infertility I think is really important.” (Provider 4, gynecological oncologist)
- “It was just so amazing to know that I still had a shred of hope. The past few weeks with chemo have been hard, but knowing that I have something in the future to look forward to gets me through it. I’m not sure it would have been this easy if I didn’t have that embryo, because I feel like, ‘What’s the point, it is not like I can have kids.’ That embryo is just the icing on the cake. It made everything so much easier.” (Survivor 1, 22 years old, White, in a relationship)
- “We thought that there was no chance for us anymore. Then they gave us this information today about possible IVF...even if we are not overly concerned about getting pregnant, it is nice to know we still have that option.” (Survivor 28, 33 years old, Hispanic, single)

**Subtheme 2: Understanding That There Are Options Is Important**

- “They need...a decision aid that says, ‘[Protecting your fertility] is an option. These are all the different options that you have. You can choose to say No. You can choose all these different routes.’” (Provider 16, breast medical oncologist)
- “I did see [the fertility specialist], and she told me there were all these other options...Just hearing the options made me feel better. I never heard options from anyone until I came here.” (Survivor 25, 22 years old, White, in a relationship)
- “For me, [a key recommendation] is ‘make sure you know all your options.’ To me the worst decision we can make is not fully understanding what all of our options are at the time.” (Survivor 6, 35 years old, White, single)

**Subtheme 3: Understanding That Family-Building Desires Change Over Time Is Important**

- “Even that person who is, OK, well, no. I’ll just take my chances, and I’m not talking to anybody - I guess my fear is that they don’t really understand what I’m saying or what they have been told, that this will probably affect their ability to have children in the future.” (Provider 3, gynecological oncologist)
- “[I recommend fertility preservation counseling] even if you are not totally certain where your life is going at the moment. I would say, while you have those resources and options available...because you may feel very differently in 5 years. I know, for me, I didn’t want kids 5 years ago and now I am kind of open to the possibility.” (Survivor 2, 32 years old, White, single)
- “I was in a relationship, but I kept postponing things, thinking, ‘35 and then I’ll start thinking about kids.’ But it is one of those things that you are not 100% sure if you can plan it.” (Survivor, 37 years old, White, single)

#### Primary Theme 2: Significant but Potentially Modifiable Barriers Exist

All participants also reported that decision-making barriers still exist, and 4 subthemes emerged (Textbox 2). The majority of survivors raised issues of awareness and time for decisions and uncertainty about risks and costs. Many confirmed that they recalled hearing about fertility in the informed consent for chemotherapy; however, less than half reported understanding fertility preservation decisions. More than half reported feeling they did not have time to consider fertility decisions or collect needed information (eg, insurance paperwork and fertility status), although several acknowledged that they were anxious to start treatment quickly. Most women reported difficulty making decisions because of costs—either because they could not afford preservation without insurance or because they did not understand these decisions early enough to have time to plan for the short- and long-term out-of-pocket costs.

**Subthemes and exemplary quotes for primary theme 2 (significant but potentially modifiable barriers exist).**

**Subtheme 1: Need for Provider and Patient Education**

- “Those are questions that are really best for...the reproductive [endocrinologist].” (Provider 7, radiation oncologist)
- “[I am] reasonably satisfied [with my fertility preservation knowledge]. I’m not an expert in that area...I provide some general counseling [and tell patients], ‘I certainly can’t deliver [personalized risks and rates of fertility preservation outcomes], but I’m pretty sure that [the reproductive endocrinologist] can.’” (Provider 16, breast medical oncologist)
- “The thing is, they didn’t tell me anything about it [chemotherapy affecting infertility], so I couldn’t say yes or no. Now I’m learning about all of this, that chemotherapy can affect it.” (Survivor 32, 23 years old, Black, in a relationship)
- “I had no idea. They didn’t tell me when I was diagnosed that this might cause problems having kids. Nobody told me that. It is still new to me. They didn’t tell me until a couple of months ago. That is why I am doing everything I can. I have been wanting kids since I was 9...always, always, always.” (Survivor 19, 21 years old, White, single)

**Subtheme 2: Limited Time for Making Decisions and Facilitating Referrals**

- “[Patients] come in on Monday, have tests on Tuesday and Wednesday, and by Friday we are starting treatment. There are times when you have to start that same day.” (Provider 10, gynecological oncologist)
- “...to go from chemo directly to fertility within that one hour is too much. So if there was...online resources [for fertility preservation education], that would help.” (Provider 16, breast medical oncologist)
- “I would say generally, less than 5 minutes. That would be my usual conversation.” (Provider 1, medical oncologist)
- “It was incredibly frustrating, because...there is not a direct path to get them a referral...your ovarian reserve declines, but we don’t really have a mechanism to actually test that routinely in patients and counsel patients with their true fertility potential.” (Provider 1, medical oncologist)
- “...you have cancer, and that’s hard enough. But then to be told that you have to make a decision right now, because we can’t delay it...[The doctor] says I can’t even think about it one night.” (Survivor 30, 33 years old, White, married)

**Subtheme 3: Cost of Fertility Preservation Services**

- “The barriers would probably be insurance. That is maybe number one; that is 100%.” (Provider 8, radiation oncologist)
- “Payment is a barrier for women but I think the ones that really want to pursue it, we find options for them.” (Provider 5, nurse practitioner)
- “...what I have been researching really has been around funding,...the cost factor, because I know that this is something that I want to do. However, [the cost] is my roadblock.” (Survivor 3, 39 years old, Black, single)
- “I was going to go through with the egg preservation...but then, like I said, once I saw the prices and everything, I was, as much as I want to do this, I think I have to accept if for what it is right now. And later on get over that when it comes...” (Survivor 25, 23 years old, Black, in a relationship)

**Subtheme 4: Cultural and Financial Differences Are Important, but They Do Not Stereotype Services**

- “I have over time learned...not to stereotype and pitch the whole thing based on ethnicity and religion...just say that these are personal decisions based on your own morals and religion, your previous experience, and your family’s experience.” (Provider 15, gynecological oncologist)
- “I guess more than anything, being mindful that there is not one cookie cutter approach, that every situation is so different.” (Provider 1, medical oncologist)

Most providers also discussed awareness, time, and cost barriers to decision making and added the need for additional education about the ASCO guidelines, updated risks of newer procedures, and the time or processes needed for fertility preservation. Many providers also discussed the need for information tailored to cancer type but viewed their role as introducing the topic and facilitating referrals to a reproductive endocrinologist for personalized decision counseling. A majority of providers expressed a need for practical support to identify appropriate women and facilitate the process (eg, navigating timely referrals and planning for the future). Notably, all oncologists reported feeling comfortable introducing treatment-related infertility; however, a few providers who expressed higher levels of knowledge and comfort also reported discussing decisions with patients more often and in greater detail.

Over half of the providers discussed the relevance of patients’ demographic characteristics (particularly age, culture, spiritual beliefs, and potential resources) for introducing the topic appropriately; however, they cautioned against making assumptions, noting that many women or families make unique decisions and find additional resources (eg, family contributions and fundraising). Seven providers brought up scenarios in which they initially felt it might be okay not to offer a fertility preservation decision aid (eg, noncurative treatment plan, single women, older age, multiple children already, low socioeconomic status, and religion). However, they quickly provided examples of exceptions (eg, a family who wants to freeze their dying daughters’ eggs or embryos and remarried mothers who strongly want more children) and emphasized offering information equally yet compassionately to all women.

#### Primary Theme 3: Support for a Carefully Designed Fertility Preservation Patient Decision Aid

All participants supported the use of a fertility preservation patient decision aid, provided it was carefully designed to overcome the barriers. Three subthemes arose in their recommendations: provide tailorable content, use appropriate and inclusive language, and design multiple delivery formats and routes (Textbox 3).

**Subthemes and exemplary quotes for primary theme 3 (strong support exists for a carefully designed patient decision aid).**

**Subtheme 1: Providing Tailorable Content**

- “The 18- to 22-year-olds may be different than the 30 year-old because the parents are going to be watching more. Split it up between minors and non-minors.” (Provider 16, breast medical oncologist)
- “[Tailor by] solid versus liquid tumor, because that would be different.” (Provider 1, medical oncologist)
- “It would be really cool if you [could] put in a patient’s age, history, results,...then it brings up their algorithm, including the known statistics about a given cancer and what their outcomes potentially are. That would help the patient and the provider.” (Provider 3, gynecological oncologist)
- “I like details. I am going to click on that and get every detail possible. I’m like that. I think to grab someone’s attention, something general, but then when you click on it, a bunch of details on that one topic.” (Survivor 2, 21 years old, White, single)
- “I think they [patient stories] are helpful, because it shows that other people have gone through the same thing and how they came to their decisions.” (Survivor 33, 24 years old, White, in a relationship)

**Subtheme 2: Using Appropriate and Inclusive Language**

- “Some countries like Qatar, have reproductive medicine and it is not a big deal (shipping international can be a barrier), but for Kuwait, Saudi Arabia, UAE, etc, it may be a big issue. Sometimes women cannot get married if they are infertile.” (Provider 6, pediatric nurse practitioner)
- “...particularly Orthodox Jewish populations and having families is very important to them. While they don’t talk about sex overtly, they are very interested in finding way to preserve fertility because that is so important to their religion. If they have concerns, it typically goes through the Rabbi.” (Provider 10, gynecologic oncologist)
- “...with Arabic women, many of them are virgins, so doing a transvaginal ultrasound or a transvaginal procedure becomes an issue. I had one patient that declined because she wouldn’t do that.” (Provider 15, gynecological oncologist)
- “We only found one company [that]...was open to same sex adoptions.” (Survivor 10, 31 years old, White, in a relationship)

**Subtheme 3: Designing Multiple Delivery Formats and Routes**

- “[New patients] need to hear it two or three different ways because they are not processing a lot in those first three visits (other than chemo and surgery).” (Provider 1, medical oncologist)
- “It could be part of the nursing intake (Are they 45 or under? Make sure you give them the fertility information to watch). I would involve nurses and midlevels.” (Provider 16, breast medical oncologist)
- “We have different education backgrounds, so sometimes people can interpret a flowchart better than others, and people might relate better to the story of this person that went through it.” (Provider 17, nurse practitioner)
- “The nurses have that way of going, ‘What else is going on?’ I think that is the perfect time to say, ‘OK, you may not be thinking about it, but these are some of the things that you may want to consider and ask.’” (Provider 1, medical oncologist)
- “The simplest thing would be if there was one piece of paper that I could just hand them and say, ‘You can go to this website and explore this decision aid, and we can discuss it next time.’ And offer periodic [group] discussions.” (Provider 9, radiation oncologist)
- “I use the [institution] website for a lot, so it is handy that it has the mobile application, and the desktop. Although they look a little bit different, they are pretty much the same. For simplicity, it is nice to have it optimized for the mobile device.” (Survivor 30, 33 years old, White, married)
- “To be honest, probably because of my age, no. I don’t like those stupid movies...I think it has to do with the fact that I have grown up and been taught to be a strong independent woman. I don’t feel like I need to lean on those types of things.” (Survivor 11, 22 years old, White, in a relationship)

Survivors’ recommendations focused on the content and ability to select the level of information and support needed. Their recommendations varied from brief introductory overviews (leaving the majority of the information for the fertility consultation) to detailed comparison charts and suggestions for addressing uncertainty, managing emotions, and navigating the financial process. The majority of survivors recommended having a way to select only their relevant options to minimize information overload. They also requested implicit decision guidance (eg, examples and testimonials about decision making) and inclusion of multiple lifestyles, cultures, and beliefs.

Providers recommended a tool that presented options tailored to clinical factors, such as age and cancer type. They also recommended providing explicit decision-making activities (eg, value clarification exercises and preference elicitation) to assist in identifying who may benefit from a referral for fertility counseling. Several providers mentioned the need for low health literacy and culturally appropriate language, and many providers cited examples of the importance of virginity and fertility in many cultures.

The majority of recommendations in both groups focused on delivery. Both survivors and providers recommended offering a patient-facing decision aid at multiple time points using multiple media, with an emphasis on viewing outside the consultation. Suggestions included providing an initial handout, booklet, or email at the first and/or second visit after diagnosis that contained a URL link to an institutionally supported website, viewable on a computer, tablet, or phone. Survivors also suggested having a nurse or a peer volunteer personally recommend the decision aid to emphasize the importance of fertility and distinguish it among the many handouts received at diagnosis. Both groups supported offering a website to allow women to view it when optimal. Notably, providers recommended web delivery to address diagnostic consultation time limitations and to enable patients to self-refer to a fertility specialist. Survivors recommended web delivery to address information overload and coping barriers. A few survivors also mentioned wanting to revisit the information after cancer treatment.

#### Additional Informative Responses

In addition to the primary themes, a few individuals raised the following four points, which the stakeholder panel recommended reporting to inform decision counseling. One provider noted the importance of clearly discussing alternative family-building options, including acknowledging the potential challenges for cancer survivors considering surrogacy or adoption. Two survivors recommended including testimonials from women at various stages of cancer treatment and survivorship. Two others mentioned addressing their partners’ preferences and support. A pediatric oncologist noted the communication barriers for young adults and their parents and suggested a pediatric version of a patient decision aid that offers tangible support:

[Tell patients:] We will help [you] talk to your mom. There are resources...our social worker may be able to help. We will talk to the oncologist.

Provider 6, pediatric nurse practitioner

### Follow-up Focus Group

Participants in the ad hoc focus group confirmed and clarified the findings of the individual interviews and deepened explanations. There was unanimous agreement that fertility should be explained as early as possible. Survivors particularly commented on needing time to make the transition from initial awareness and comprehension to delving into the potential costs, insurance processes, ethical considerations, and future decisions. A few women reiterated needing to acknowledge that the "new normal" may include uncertainty when making decisions.

Women related several examples of feeling rushed, unaware, or pressured, which led to unanswered questions and unspoken preferences, with continued feelings of regret and sadness. Several mentioned feelings of reliving the trauma as survivors and "not being able to own that decision." Several comments were made regarding the need to include fertility preservation in their financial planning and acknowledging resources other than insurance, such as foundations, family contributions, and social fundraising.

During the review of the cancer center’s patient information handouts, existing decision aid materials from other countries [5,28,46,47], and additional resources identified by interview participants, survivors appreciated the explanations of the process or timeline, examples or testimonials, and directly addressing fears about time. They also noted the importance of a "gentle" and "compassionate" tone. Survivors felt that some of the tools seemed outdated or would not be applicable across the diverse population in this health system. They also requested information for other types of cancer and optional sections discussing referrals, insurance coverage, and estimated costs. Finally, they also supported web delivery to provide links to financial resources and fertility specialists.

Focus group participants also brought up additional topics that they thought should be included in decision support materials, such as how to be your own personal champion, resolve family conflict or pressure, engage your partner in this decision, and have someone come with you to take notes and raise questions:

I kind of grieved it when I was first diagnosed with cancer, and for 7 years, kind of held my breath. [My husband] came into my life, and there was a real sad time after about a year of marriage where I was having to face it again.

Survivor 30, 33 years old, White, married

I was told [that I had cancer] on a Wednesday, by Thursday I was getting my port, and Friday and Saturday I started my treatment. All I was thinking at that time was, “Okay, do what you need to do.” So my sister was there and she said, “Wait, wait, hold on, we have to discuss this. There are other options.”

Survivor 3, 39 years old, Black, single

My mom and my boyfriend at the time were just, “We want you to be okay.” But I wanted to have my own baby...one doctor kind of yelled at me, “You need to just survive it!” I said, “If I survive, I want to be able to have babies!”

Survivor 6, 24 years old, Asian, single

## Discussion

### Principal Findings

Overall, providers and survivors emphasized the importance of fertility preservation decision making for high-quality comprehensive cancer care. Both groups acknowledged existing barriers, such as awareness, time, and costs, and proposed potential solutions. All participants strongly supported offering women a website so that they could review the material at home. They recommended providing a lay language overview, interactive features to self-tailor (eg, by cancer type), guidance in decision making about seeking or accepting a fertility referral, and links to facilitate referrals and access financing programs. They noted the value of a patient decision aid for engaging women in the initial decision and the potential for this engagement and empowerment to improve hope during the treatment process and decrease downstream regret.

### Comparison With Prior Work

These results confirm the results of previous studies regarding the multilevel challenges to providing high-quality fertility preservation counseling [8,14-16,29,48,49]. Providers and survivors noted patient-level barriers such as information overload, misinformation, coping needs, lack of awareness or knowledge, and difficulty understanding and personalizing complex medical information. They also described provider-level barriers (eg, familiarity and perceived role in the decision) and system-level barriers (eg, limited time, opportunities, and access). As in other studies, providers and survivors noted the desirability of patient stories to convey experiential knowledge; however, the potential for social matching bias (ie, attending more or less to information from someone who is more or less similar to you) remains a concern [16,50,51]. Furthermore, these findings complement the extant literature describing the tension between concerns about information overload while ensuring an informed decision-making process [52,53]. Providing accessible information that meets health literacy needs (eg, plain language, culturally aware, and relevant) may help women feel more supported and engaged in the decision-making process [52,53].

This study adds to the literature user-generated design recommendations, such as providing understandable medical information; explicitly addressing uncertainty; and helping personalize the information by providing estimates of costs, descriptions of the treatment or recovery processes, values clarification exercises, and example stories modeling decision making [29]. Providers and survivors also recommended multilevel information, literacy or numeracy support, responsive design (for use on tablets, cellular phones, etc), web delivery, and the ability to self-tailor by cancer type and cultural characteristics. Both groups described a tool that helped women prepare for a fertility consultation, emphasizing that the tool is not a replacement for a reproductive endocrinologist who can provide fertility testing and personalized counseling.

Notably, providers and survivors expressed a need for clear cost information. Providers stated that they rarely discussed costs, whereas survivors stated that costs played a significant role in their decision—either they assumed their insurance would not cover the treatments or the fertility counseling or they could not locate cost information in time to make an informed decision. The pressure to make these decisions with incomplete cost information may be forcing women to forego fertility counseling and fertility preservation treatments they need and desire. This suboptimal decision process may, in turn, compound long-term decisional regret because of a sense of unnecessary loss [54,55]. Future studies may wish to test whether a patient decision aid that includes cost estimates decreases decisional conflict and long-term regret.

Consistent with the distributions of survivors seen at the Oncofertility Clinic, this study recruited 24% non-White and 15% Hispanic women. However, a larger question remains as to whether these distributions represent optimal equitable care. Previous studies document variations in utilization of fertility preservation by race and gender and note a variety of barriers, including awareness, access, and insurance coverage [8,10,12,15,17,56-58]. We are conducting a parallel study focusing on culturally relevant features of fertility patient decision aids in Spanish and Arabic and exploring the degree to which variations are unwarranted or warranted based on cultural differences in informed patient preferences.

These results emphasize that fertility preservation decisions are as unique as the women who face them and support the ethical imperative of the guidelines to offer fertility counseling to all women who are interested or unsure [6,16,59,60]. Fertility preservation patient decision aids may be purposefully designed to raise awareness, address misconceptions, elicit informed preferences, and help women prepare for discussion. The primary goal would therefore be high-quality decision-making process and *appropriate referral*, defined as a referral that aligned with women’s values and preferences once they were informed of the infertility risk and availability of fertility preservation and alternative family-building options.

### Limitations

Providers and survivors were recruited from a comprehensive cancer with a reproductive endocrinologist; decision support needs may differ for community clinics that refer to private fertility centers. The focus group may have increased reflexivity; however, it allowed us to confirm data interpretations and explore emergent questions. For ethical reasons, we chose to interview survivors instead of newly diagnosed patients, but their wisdom provided an additional benefit—they generated a list of common *Myths and FAQs* (frequently asked questions), which will be incorporated into the planned patient decision aid. Data on childbearing preferences at diagnosis were not collected in this study; however, the results indicate that affective forecasting may be a topic to address within a patient decision aid. In addition, the findings indicate tensions and potential inconsistencies that we cannot explain within these data. Studies are needed that focus specifically on the rates and quality of shared decision-making discussions.

### Conclusions

Providers and patients continue to report unmet needs regarding timely access to fertility preservation education and decision support. Survivors emphasize the importance of explicitly addressing fertility preservation early to allow them time to make decisions and plan financially. Providers emphasize designing tools to facilitate timely information and appropriate referrals and to improve adherence to guidelines. Both women and providers supported the development of a patient decision aid website to address these challenges and recommended interactive, self-tailoring features. Providing an accessible, tailored, and meaningful patient decision aid may increase awareness, decision making, and referrals; reduce regret; and improve long-term survivorship outcomes.

## Appendix

Multimedia Appendix 1 Survivor and provider interview guides.

Multimedia Appendix 2 Focus group discussion guide.

## Abbreviations

ASCO: American Society of Clinical Oncology
FAQ: frequently asked question
IPDAS: International Patient Decision Aid Standards
