# Supplementary material for: Patients’ and Providers’ Needs and Preferences When Considering Fertility Preservation Before Cancer Treatment: Decision-Making Needs Assessment
Source: JMIR Form Res. 2021 Jun 7;5(6):e25083. doi: 10.2196/25083 (PMC8218210; doi:10.2196/25083)
Supplement: Multimedia Appendix 1 [file formative_v5i6e25083_app1.docx]

## Patient Interview Guide

### Introduction

*Moderator Instructions: After turning on the audio recorder: Please briefly re-introduce yourself, the purpose and procedures of the interview, invite questions, and confirm informed consent. Then proceed with the following discussion prompts [note: when needed, tailored prompts for providers are in brackets].*

### Current Experiences and Key Factors:

- Tell me about your experience discussing the possibility that the cancer treatments could impact fertility.
- What types of things were you thinking about when deciding whether to go see the fertility specialist (if needed: getting more information, finding out if you were fertile, costs, anything else?) [How do you decide who to refer and do you discuss any factors with patients before referring (for example, costs, timing, etc.)?]
- Did you get a referral to discuss your options for fertility preservation? How (if needed: did you talk with your oncologist or call yourself)? Tell me about how it went. [What is your referral process?]
- Previous patients tell us they often weren’t aware or had trouble finding information in time. Please tell me about your experience. Where did you go for information and why? [What information resources do you provide or recommend for women and why?]
- Did you use any of the resources here at the hospital (Patient Education, your Buddy, the MyMDAnderson network)? Did you talk with your partner, family, or friends?
- What about websites, social media, news?

### Unmet needs

- When and how did you make your decision about whether to do fertility preservation?
- Did you feel like you had enough information about the options and their risks and benefits?
- What things were most important to you when you made your decision?
- Were you thinking about cost at all? What else do you think would be helpful for women?
- Is there anything that you would have wanted to learn before making your decision?
- Tell me about who you discussed the decision with, if anyone, and what that meant to you (if needed: did you want them more/less involved, did you feel supported/pressured, did that affect your relationship)

### Recommendations

- Ok, now let’s focus on what we can do to improve this process for women. As you know, we’re hoping to create a tool that provides all women diagnosed with cancer with timely, trusted information that is understandable, useful, and meaningful. The purpose is to raise awareness, help women become well-informed about their options, help them get referrals for fertility counseling if they want one, and overall to make the process easier and more meaningful. Thinking back on your experience, please tell me about what worked well and what you would recommend we improve.
- What type of tool would you recommend (video, booklet, website, personal worksheet) and why?
- When would you recommend we give it to women and why?
- What do you think are the essential facts that we should include?
- How you would describe what fertility preservation means in your own words?
- What should we include to help women think about these decisions?
- What would be helpful for improving communication and making sure women are able to get the referral and/or treatment, if they want them?
- Is there any particular style or format or tone you would recommend to make sure it’s appropriate and inviting?
- Is there anything else we should include that you think would be helpful for women who are just diagnosed?

### Closing

- Ok, thinking back on all the questions, is there anything else you want to add?

*Moderator Instructions: Stop the audio recorder. Thank the survivor/provider for their time and for sharing their experiences and advice. Ask if they have any questions. Ensure that they receive the parking validation and gift card. Complete the participation log and moderator notes, below.*

*Participant ID: ______________ Date: ______________________________ Moderator Initials: ___________________*

*Moderator Notes (observations, tone, body language, contextual clues to inform interpretation, etc.):*

## Provider Interview Guide

### Introduction

*Moderator Instructions: After turning on the audio recorder: Please briefly re-introduce yourself, the purpose and procedures of the interview, invite questions, and confirm informed consent. Then proceed with the following discussion.*

### Current Experiences and Key Factors:

- Tell me about your experience discussing the possibility that the cancer treatments could impact fertility.
- What factors do you consider and/or discuss with patients to decide whether to refer for fertility (for example, costs, timing, etc.)?
- What is your referral process?
- What information resources do you provide or recommend for women and why?
- Did you use any of the resources here at the hospital (Patient Education, your Buddy, the MyMDAnderson network)?
- What about websites, social media, news?

### Unmet needs

- When and how do you make decisions with patients about fertility preservation?
- Did you feel like you and your patients have enough information about the options and their risks and benefits?
- What things do you think are most important when making these decisions?
- What, if anything, do you discuss about cost in these decisions?
- What else do you think would be helpful for women?
- Is there anything that you think women are missing before making these decisions?

### Recommendations

- Ok, now let’s focus on what we can do to improve this process for women. As you know, we’re hoping to create a tool that provides all women diagnosed with cancer with timely, good information that is understandable, useful, and meaningful. The purpose is to raise awareness, help women become well-informed about their options, help them get referrals for fertility counseling if they want one, and overall to make the process easier and more meaningful. Thinking about yours experiences discussing referrals for fertility counseling, please tell me about what worked well and what you would recommend.
- What type of tool would you recommend (video, booklet, website, personal worksheet) and why?
- When would you recommend we give it to women and why?
- What do you think are the essential facts that we should include?
- How you would describe what fertility preservation means in your own words?
- What should we include to help women think about these decisions?
- What would be helpful for improving communication and making sure women are able to get the referral and/or treatment, if they want them?
- Is there any particular style or format or tone you would recommend to make sure it’s appropriate and inviting?
- Is there anything else we should include that you think would be helpful for women who are just diagnosed?

### Closing

- Ok, thinking back on all the questions, is there anything else you want to add?

*Moderator Instructions: Stop the audio recorder. Thank the survivor/provider for their time and for sharing their experiences and advice. Ask if they have any questions. Ensure that they receive the parking validation and gift card. Complete the participation log and moderator notes, below.*

*Participant ID: ______________ Date: ______________________________ Moderator Initials: ___________________*

*Moderator Notes (observations, tone, body language, contextual clues to inform interpretation, etc.):*
