# Supplementary material for: Patients’ and Providers’ Needs and Preferences When Considering Fertility Preservation Before Cancer Treatment: Decision-Making Needs Assessment
Source: JMIR Form Res. 2021 Jun 7;5(6):e25083. doi: 10.2196/25083 (PMC8218210; doi:10.2196/25083)
Supplement: Multimedia Appendix 2 [file formative_v5i6e25083_app2.docx]

## Focus Group Discussion Guide

Framework Informed Consent [3 minutes]

Re-introduce the purpose of the focus group, consent information, etc.

Re-iterate confidentiality: Please do not repeat anything said in this room after the focus group concludes.

Baseline Questionnaire & Decision-making Values Statements [7 minutes]

Before we begin the focus group, please take a few minutes to fill out the following questionnaire. On the worksheet, please rank the statements in order of how important they were to you when you made your decision whether or not to pursue fertility preservation.

Introductions [4 minutes]

We will begin recording in just a moment. Before we do, does anyone have any questions or comments?

*Begin recording.*

Now, let’s go around the room and introduce ourselves to the group. You can tell us your diagnosis and what you decided to do for fertility preservation, if anything. Also let us know something about yourself other than your experience with cancer. You can include as much or as little information as you’d like (for example, where you’re from, what you do for work, favorite restaurant, etc.)

Discussion of the Decision-making Values Statements [20 minutes]

- Would anyone like to share why you ranked the statements the way you did?
- Was it tough to choose the most important statement? Why or why not?
- What other rankings were difficult to decide between? Why?
- What was the least important statement to you? Why did this not matter as much to you?
- Was there anything missing from this list? If so, what?
- [For topics that weren’t mentioned] I noticed that _______ wasn’t mentioned by anyone. Did anyone find this important? If not, why not?
- [If anyone hasn’t spoken yet, ask them what they think was most/least important.]

Materials Review

Now we are going to review some materials that may or may not be familiar to you. Not all these materials may be relevant to your type of cancer or your fertility preservation choice, but please review them anyway. While reading, think about what you find helpful, what you think needs more information, or what would help you understand the material better.

*YSC FP Materials [8 minutes reviewing, 10 minutes discussing]*

- Did you find these 3 pages helpful to understand fertility preservation? Why or why not?
- What did you think about:
  - The amount of text?
  - The level of detail?
  - The image of the family?
  - The colors used? (Show them the color version)
- What do you think the most important part of this reading was?
- Put yourself in the shoes of a woman who was recently diagnosed with breast cancer. What do you think you would be most interested in in this section?
- If these materials were put online, what could or should be added to make it more engaging/clear/informative/helpful (eg, videos, pop-up definitions, links to outside sites, etc.)?

*Fertile Hope Materials [5 minutes reviewing, 5 minutes discussing]*

Many of you may have already seen this pamphlet from the Livestrong Foundation.

Even if you have, please everyone take a few minutes looking over the pamphlet. You don’t have to read everything. We are more interested in what you think about its format and layout.

- For those of you who have not seen this pamphlet before, what were your initial thoughts about it? Trustworthy? Professional? Useful? Informative? What makes you say that?
- Would you have read through all the material if this was given to you at your fertility preservation consultation? Why or why not?
- For those of you who have seen this before, did you use it? If so, how? If not, why not?
- The website is mentioned in the pamphlet. Did anyone go to the site because of this pamphlet? Would you have? Why or why not?
- Were there other websites you went to when you were thinking about fertility preservation?

*Tendrils Questions to Ask Yourself [3 minutes reviewing, 7 minutes discussing]*

Take a couple of minutes to review the questions on the worksheet provided.

- Are these the types of questions that you asked yourself when you were decided whether or not to pursue fertility preservation?
- If so, what did you find helpful when you were answering these questions for yourself?
- If not, what types of questions did you ask yourself?
- Do you wish you had asked questions like these when you were considering FP?

*Internet/Websites [10 minutes]*

- When you use the Internet, what device do you usually use? Does it depend on what type of website you’re going to? What are some of your favorite websites to go to?
- Do you like the layout of these example websites? What is it about the layout that you like?
- What are some things that annoy you about certain websites (eg, broken links, hard navigation, colors)?

Recommendations for a Web-based Decision Aid [10 minutes]

As you know, we are creating a decision aid to help women with cancer decide if they want to pursue fertility preservation. Now we are going to ask you some questions to help us decide different features about the decision aid.

We are planning to have the decision aid available online.

- How comfortable would you be going online to use a decision aid?
- Would you print out materials from the decision aid to take to your doctor?
- Should print materials also be provided to women considering fertility preservation? If so, how should they look? (Pamphlets, spirals, handouts, etc.)
- Who would you want to discuss the decision aid with (eg, your oncologist, a fertility specialist, nurse)? Why?
- Would you want to go through a decision aid with your: Family? Friends? Partner/spouse?

We are thinking about using certain features in the decision aid. Would you like the following:

- Video interviews with women who have done fertility preservation
- Exercises that help you think about your values (like in the first questionnaire)
- Videos or pictures of fertility preservation techniques

Regrets/Advice for Other Women [5 minutes]

- As we finish our conversation, is there anything we have talked about that you wish you had considered when you were making your decision?
- Do you have any regrets about the choice you made?
- How important was this decision in comparison to the other decisions you had to make when you were diagnosed with and were treating your cancer?
- How important is it for women to consider fertility preservation before they undergo cancer treatment?
- Finally, what words of advice would you give a woman who is considering fertility preservation?

*End recording.*

*Thank everyone for time and effort. Ask for them to sign and print their names on the receipts inside the envelopes. Make field notes as needed.*
